# Exploiting Shock Waves to Trigger the Anticancer Sonodynamic Activity of 5-Aminolevulinc Acid-Derived Protoporphyrin IX on In Vitro 2D and 3D Cancer Models

**DOI:** 10.3390/biomedicines10030615

**Published:** 2022-03-06

**Authors:** Federica Foglietta, Patrizia Panzanelli, Loredana Serpe, Roberto Canaparo

**Affiliations:** 1 Department of Drug Science and Technology, University of Torino, Via Pietro Giuria 13, 10125 Torino, Italy; federica.foglietta@unito.it (F.F.); roberto.canaparo@unito.it (R.C.); 2 Department of Neuroscience Rita Levi Montalcini, University of Torino, Via Cherasco 15, 10126 Torino, Italy; patrizia.panzanelli@unito.it

**Keywords:** shock waves, sonodynamic therapy, 5-aminolevulinc acid, protoporphyrin IX, three-dimensional cancer models

## Abstract

Sonodynamic therapy (SDT) is a noninvasive method for cancer treatment based on selective activation of a sonosensitiser by ultrasound (US), which results in the generation of reactive oxygen species (ROS) and cancer cell death. SDT uses a similar approach to photodynamic therapy (PDT), but can overcome the main drawback of PDT, i.e., poor tissue penetration of light. This research work investigated the anticancer effect of SDT on various two- (2D) and three-dimensional (3D) in vitro tumour models, using PDT as a reference treatment. Sonodynamic experiments were performed with pulsed US, specifically with shock waves (SW) and the prodrug 5-aminolevulinic acid (Ala), which is converted—at the mitochondrial level—into the sonosensitiser protoporphyrin IX (PPIX). SW-mediated PPIX sonodynamic activation resulted in a significant decrease in cell proliferation, especially on human fibrosarcoma (HT-1080) cells, where PPIX accumulation was higher compared to human melanoma (A2058) and neuroblastoma (SH-SY5 Y) cells. Moreover, SW-mediated SDT showed significant ROS generation, cell line-dependent in its amount, probably due to differences in Ala-induced PPIX synthesis. In all cancer cell lines, apoptosis was highlighted as the main cancer cell death pathway determined by SW-mediated SDT, along with significant cytochrome c release, and a consequent increase in DNA damage. The efficacy of SDT with SW and Ala in halting cancer cell proliferation was also confirmed in 3D cancer spheroids. The present study suggests that SW-mediated SDT is a valuable approach to slow down tumour proliferation, thus opening an innovative scenario in cancer treatment.

## 1. Introduction

The biomedical applications of ultrasound (US) have evolved from its ubiquitous clinical use as an imaging tool to include an expanding array of therapeutic capabilities for treating cancer [1]. Interestingly, the association between US and a chemical responsive agent (sonosensitiser) in the so-called sonodynamic therapy (SDT) represents a noninvasive anticancer approach that exploits US energy [2,3]. Specifically, the energy released by US-induced collapsing bubbles, in the phenomenon of inertial cavitation, generates high temperature and pressure which provide adequate conditions for pyrolysis, generating reactive oxygen species (ROS) such as hydroxyl radicals (·OH) [4]. Moreover, the bubble–liquid interface generates strong temperature and pressure gradients that can disintegrate the surrounding solutes, and the energy released during the bubble collapse can provoke chemical reactions in the surrounding solution. The relationship between ROS formation and cavitation has been extensively studied, allowing researchers to associate inertial cavitation to a progressive sonoluminescence generation with sonosensitiser-mediated ROS generation [5,6,7,8,9]. Even if the effectiveness of SDT has been demonstrated in several tumour models, there is still limited knowledge of the specific mechanism of interaction between the sonosensitiser and US in tumour tissues, although it seems that inertial cavitation plays a crucial role. In these terms, the peak wave pressure of US and the US insonation technique represent the most important parameters recruited to generate inertial cavitation. 

To this end, the use of pulsed US such as shock waves (SW) to activate the sonosensitiser is a valid strategy to minimise the thermal effects, enhancing the nonthermal effects associated with acoustic cavitation [10,11]. 

Shock waves are sharp discontinuities, characterised by a peak positive pressure (between 30 and 150 MPa) with a phase duration between 0.5 and 3 μs, followed by a tensile wave with a peak negative pressure that drops to 20 MPa with a duration of 2–20 μs [12]. This decrease in pressure causes the cavitation phenomenon. For several years, SW have been extensively used in lithotripsy where stone diseases are treated in patients in a noninvasive way [7]. Recently, the use of SW with proper parameters to activate sonosensitisers has been investigated not only in vitro but also in vivo [10,13,14,15], showing the potential of SW to boost the efficacy of SDT as a valid and innovative way for treating cancer. 

Another important aspect in the evaluation of a new therapeutic approach is the model to be used. Conventionally, the two-dimensional (2D) cell culture, where cells are grown as monolayers on a flask surface, has a central role in research, but shows many limitations due to the 2D model inaccuracy of representing tissue cells in vitro [16]. On the other hand, the three-dimensional (3D) cell culture has demonstrated several enhancements in several studies targeted towards morphology, cell number proliferation and monitoring, differentiation, response to stimuli, protein synthesis and drug metabolism. All of this is made achievable by the ability of the 3D culture to resemble in vitro a more realistic representation of in vivo features [17,18]. As it is established that different morphological and cellular features of tumour cells can influence the therapeutic results [19], in this study, we considered three different human cancer cell lines, namely melanoma, fibrosarcoma and neuroblastoma, grown as 2D and 3D cell cultures, in order to investigate the SDT anticancer activity, by combining SW with the sonosensitiser protoporphyrin IX (PPIX) derived from the mitochondrial metabolisation of the prodrug 5-aminolevulinc acid (Ala). Moreover, to strengthen our work, we took PDT as the reference treatment.

## 2. Materials and Methods

### 2.1. 2D and 3D Culture of Cell Lines

The A2058 human melanoma cell line, obtained from the American Type Culture Collection (ATCC, Rockville, MD, USA), was cultured in Dulbecco’s modified Eagle’s medium (DMEM) supplemented with 2 mM L-glutamine, 100 UI/mL of penicillin, 100 μg/mL of streptomycin plus 10% foetal bovine serum (Sigma Aldrich, St. Louis, MO, USA). The Minimum Essential Medium Eagle (MEME) supplemented with 100 Ul/mL of penicillin, 2 mM L-glutamine, 100 μg/mL of streptomycin plus 10% foetal bovine serum (Sigma Aldrich, Milano, Italy) was used to culture HT-1080 human fibrosarcoma cells (ATCC). The SH-SY5 Y human neuroblastoma cell line, obtained from the Interlab Cell Line Collection (ICLC, Genova, Italy), was cultured in 50% F-12 and 50% Minimum Essential Medium Eagle (*v/v*) (MEME) supplemented with 100 UI/mL of penicillin, 2 mM L-glutamine, 10% foetal bovine serum and 100 μg/mL of streptomycin (Sigma Aldrich, Milano, Italy). All cell lines were maintained at 37 °C in a humidified atmosphere of 5% CO_2_ air. For all cell lines, 3D spheroids were prepared using Perfecta3D^®^ 96-Well Hanging Drop Plates (3D Biomatrix, Ann Arbor, MI, USA). Briefly, cells were detached and counted; and 5000 cells per well for A2058 and HT-1080 cells, and 5500 cells per well for SH-SY5 Y cells were used and seeded in 40 µL of media into each well of the plates. Spheroids were left to grow for 4 days before performing sonodynamic treatments, and the cell medium was changed every 2 days.

### 2.2. Glutathione Intracellular Concentration

The total glutathione (GSH) intracellular concentration in A2058, HT-1080 and SH-SY5 Y cells was obtained using the Glutathione Assay Kit (Sigma Aldrich, Milano, Italy), according to the manufacturer’s instructions. The GSH content (nmol) was normalised to cell number (1 × 10^6^ cells) in each sample. A calibration curve was performed by using a standard curve with two-points. Briefly, GSH reacts with 5,50-dithiobis (2-nitrobenzoic acid) (DTNB) to produce GSSG and the 1,3,5-trinitrobenzene (TNB) anion, detected by absorbance. GSSG, reduced by the enzyme glutathione reductase, releases GSH, which make a reaction with another molecule of DTNB. Furthermore, the rate of TNB production is measured compared to a single determination of how much DTNB reacts with GSH, which is proportional to the initial amount of GSH. Finally, a microplate reader Asys UV 340 (Biochrom, Cambridge, UK) was used to read the plate at 412 nm, and the GSH amount was expressed in nmol/1 × 10^6^ cells.

### 2.3. Evaluation of Intracellular Ala-Derived PPIX 

The intracellular amount of Ala-derived PPIX was evaluated by flow cytometry (Accuri C6 Cytometers, Milano, Italy) at an excitation of 488 nm, after cell exposure to Ala (from 0.45 to 3.0 mM in A2058 and HT-1080 cells, and from 0.45 mM to 3.6 mM in SH-SY5 Y cells for 12, 4, and 24 h, respectively). The time for each cell line for Ala incubation was chosen based on previous observations in cells (data not shown). Intracellular PPIX was shown as the integrated median fluorescence intensity (iMFI), which indicates the product of the frequency of PPIX-positive cells and the median fluorescence intensity of the cells. The most appropriate Ala concentration for the study was then selected based on the iMFI ratio, representing the difference in the iMFI of treated and untreated cells over the iMFI of untreated cells. To visualise the increased concentration of intracellular PPIX after Ala administration, confocal image analysis was performed. Briefly, 6.0 × 10^4^ A2058, 3.0 × 10^4^ HT-1080 and 8.0 × 10^4^ SH-SY5 Y cells were left to attach for 48 h on glass coverslips in 24-well plates and then incubated with cellular medium containing a noncytotoxic concentration of Ala (0.45 mM) in A2058 cells for 12 h and in HT-1080 cells for 4 h, while SH-SY5 Y cells were incubated with Ala 1.8 mM for 24 h. At the end of incubation, cells were washed with PBS (Sigma Aldrich, Milano, italy) and fixed with paraformaldehyde 4% (Sigma Aldrich, Milano, Italy). Finally, the coverslips were mounted onto a glass slide and immunofluorescence images were acquired by laser scanning confocal microscopy, using a 40× lens, NA 1.4, 1024 × 1024 pixels (Zeiss LSM 700, Jena, Germany).

### 2.4. In Vitro Sonodynamic and Photodynamic Treatment

Sonodynamic treatment was performed using a piezoelectric SW generator (Piezoson 100, Wolf) to generate focused SW. In detail, A2058 cells were treated with SW at an energy flux density (EFD, i.e., the energy at the focal point, recorded as mJ/mm^2^) of 0.32 mJ/mm^2^ for 1000 impulses at 4 impulses/s, (SW1), while HT-1080 and SH-SY5 Y cells were treated with 0.43 mJ/mm^2^ for 500 impulses at 4 impulses/s, (SW2). An evaluation of EFD parameters was performed on cell growth to determine the correct parameters able to induce the sensitiser activation and, at the same time, not able to induce damage to cells alone.

Common US gel was applied between the treated well and the device. Briefly, regarding the 2D cell culture, A2058, HT-1080 and SH-SY5 Y cells in the exponential phase were preincubated in the dark with culture medium containing different Ala concentrations of, respectively, 0.45 mM for 12 h in A2058 cells, 0.45 mM for 4 h in HT-1080 cells and 1.80 mM for 24 h in SH-SY5 Y cells. Following incubation, cells were detached from the flask using 0.05% trypsin-0.02% EDTA solution (Sigma Aldrich). Then 5 × 10^5^ cells—resuspended in 0.4 mL of Phosphate-Buffered Saline (PBS)—were seeded into 96-well plates and treated with SW. Regarding the 3D cell culture, on day 4 of spheroid growth, selected spheroids of each cell line were pre-incubated in the dark with Ala (0.45 mM for 12 h in A2058 cells, 0.45 mM for 4 h in HT-1080 cells and 1.80 mM for 24 h in SH-SY5 Y cells). Following incubation, spheroids were washed three times with PBS and then treated with SW. The spheroid growth rate was monitored by determining the diameter of each spheroid using the Leica Application Suite Software (Leica SLF 4000 microscopy, Wetzlar, Germany), and the volume (V), expressed as V = 4/3πr^3^, was calculated at 24, 48 and 72 h after SDT with Ala in all cell lines.

For the PDT, at the end of each incubation, cells were detached from the flask with 0.05% trypsin-0.02% EDTA solution (Sigma Aldrich, Milano, Italy) and 5 × 10^5^ cells were resuspended in 1 mL of PBS into polystyrene tubes. A specific custom compact system for PDT was used, characterised by a light diffuser all-around cell culture and fixed source-cuvette positioning. The light-emitting source of the system was a LED light with a maximum radiant power of 20 mW (emitted flux), which had a central wavelength of 405 nm. A continuous radiant flux regulation from 0 to 20 mW with a nonswitching diode programmable current source was therefore used for the system. The energy fluency rates of the light radiation were adjusted to 15 mW/cm^2^ for 5 min. Moreover, all the in vitro PDT was performed in the dark.

### 2.5. Cell Proliferation Assay

Effect on cell proliferation was assessed by WST-1 assay (Roche Applied Science, Penzberg, Germany) at 24, 48 and 72 h after SDT or PDT with Ala (0.45 mM in A2058 and HT-1080 cells, 1.80 mM in SH-SY5 Y cells). Briefly, after the various treatments, 1.25 × 10^3^ A2058 and HT-1080 cells, and 2.5 × 10^3^ SHSY5 Y cells were seeded in 100 μL of culture medium in replicates (*n* = 8) in 96-well culture plates (TPP). WST-1 reagent (10 μL, Sigma Aldrich) was added at 24, 48 and 72 h and the plates were incubated at 37 °C in 5% CO_2_ for 1.5 h for A2058 and HT-1080 cells, and for 3.5 h for SH-SY5 Y cells. The well absorbance was measured at 450 and 620 nm (reference wavelength) in a microplate reader (Asys UV340, Biochrom, Cambridge, UK).

### 2.6. Flow Cytometric Analyses

Cell death analysis was evaluated by flow cytometry (Accuri C6, Milano, Italy) with SYTOX Green and APC-Annexin V kit (Invitrogen, Milano, Italy). Apoptosis was evaluated in HT-1080 and SH-SY5 Y cells at 24 h after SDT and PDT, and in A2058 cells at 48 h after SDT and PDT, according to cell proliferation data. Briefly, at the end of each treatment, cells were detached with trypsin and washed with PBS at 1500 rpm for 5 min. Cell pellets were then resuspended with 1 x Annexin-binding buffer and stained with APC-Annexin V and SYTOX Green. Sample analyses were performed at 630 nm to measure APC-Annexin V and at 488 nm excitation to measure SYTOX Green. A total of 10,000 events were considered in the analyses and cell debris with low forward light scatter (FSC) and side light scatter (SSC) was excluded from the analyses. Apoptotic cells (APC-Annexin V positive) and necrotic cells (APC-Annexin V and SYOTX Green positive) were distinguished from viable cells (APC-Annexin V and SYTOX Green negative). All analyses were carried out using FCS Express software version 4 (BD Bioscience, Milano, Italy).

ROS production was evaluated by 2,7-dichlorofluorescein diacetate (DCF-DA, Molecular Probes, Carlsbad, CA, USA) through flow cytometric analysis (Accuri C6, Milano, Italy) at 1, 5, 30, and 60 min after SDT or PDT. The amount of ROS produced was therefore expressed as the iMFI, which indicates the frequency product of ROS-producing cells and the cell median fluorescence intensity. The iMFI ratio was estimated to generate and understand the fluorescence increase at each time point [20].

### 2.7. Cytochrome c Analysis

Cytochrome c was analysed at 24 h after SDT and after PDT in all cell lines, according to cell death data. Cytosolic cytochrome c was detected in supernatants from cells selectively permeabilised by an enzyme-linked immunosorbent assay (eBioscience, San Diego, CA, USA). Briefly, cells from each condition were collected by trypsin, lysed with Lysis Buffer and centrifuged and then supernatants were analysed according to the manufacturer’s instructions. The absorbance of each well was obtained at 450 nm, with a reference wavelength at 620 nm in a microplate reader (Asys UV340, Biochrom, Cambridge, UK). A standard curve based on reference standards was used to calibrate cytochrome c concentration (ng/mg of protein).

### 2.8. Evaluation of DNA Damage

DNA damage was evaluated at 24 h after SDT using the DNA damage-AP site-Assay kit (Abcam Company, Burlingame, California), according to cell death data. Briefly, to evaluate oxidative DNA damage, an aldehyde reactive probe (ARP) was used, thanks to ARP reaction with aldehyde groups resulting from protein or DNA modification. Aldehyde sites in both protein and DNA can be converted to biotin-tagged aldehyde sites, detected by avidin-conjugated reporters, by using an excess amount of ARP. Briefly, 5 × 10^5^ cells were treated, 24 h after SDT all samples were analysed following the manufacturer’s instructions. Samples were analysed on the flow cytometer (Accuri C6, Milano, Italy) at 488 nm to detect and measure avidin signals. Cell debris characterised by low FSC and SSC were excluded from the analyses, considering a total of 10,000 events.

### 2.9. Real-Time RT-PCR

At 24 h after SDT and PDT treatments, total RNA was isolated from all cell lines. A RNA cell protection reagent (Qiagen, Milano, Italy) was used to collect cells, which were maintained at −80 °C. The RNeasy^®^ Plus Mini Kit (Qiagen) was used to determine total RNA. Moreover, the Quant-iT™ RNA Assay Kit (Invitrogen) was used to determine total RNA concentration (μg/mL) by using the Qubit (Invitrogen) fluorimeter; moreover, according to the manufacturer’s instructions, a calibration curve was created by using a two-point standard curve. The total RNA 6000 Nano Kit (Agilent Technologies, Milano, Italy) was used to determine the RNA integrity from all samples considered using the Agilent 2100 Bioanalyzer (Agilent Technologies). Total RNA (500 ng) was considered for the real-time reverse transcription (RT)-PCR and reverse-transcribed in a 20 μL cDNA (complementary DNA) reaction volume, thanks to the QuantiTect^®^ Reverse Transcription Kit (Qiagen); moreover, each 10 μL real-time RT-PCR reaction contained 12.5 ng of cDNA. The master mix used in the quantitative RT-PCR was the SsoFast™ EvaGreen (Bio-Rad, Milan, Italy), and the QuantiTect Primer Assay (Qiagen) was used as the gene-specific primer pair for *APAF1* (Cat No QT00092358), *BAX* (Cat No QT00031192) and *BCL-2* (Cat No QT00025011). The transcript of the reference gene ribosomal 18 s and 28 s RNA (*RRN1*) was used to normalise mRNA data. A MiniOpticon™ Real-Time PCR system (Bio-Rad) was used for RT-PCR by using a specific PCR protocol with the following conditions: HotStarTaq DNA polymerase activation step at +95 °C for 30 s, followed by 40 cycles at +95 °C for 5 s and +55 °C for 10 s. In all PCR runs, at least three independent cDNA preparations per sample were used along with a duplicate run for all samples, including two nontemplate controls. Data analysis was performed by the Bio-Rad CFX Manager Software version 3.0 (Bio-Rad), in conformity with the Minimum Information for Publication of Quantitative Real-time PCR Experiments (MIQE) guidelines [21].

## 3. Results

### 3.1. Determination of Glutathione Intracellular Levels in A2058, HT-1080 and SH-SY5 Y Cells

**Figure 1:**
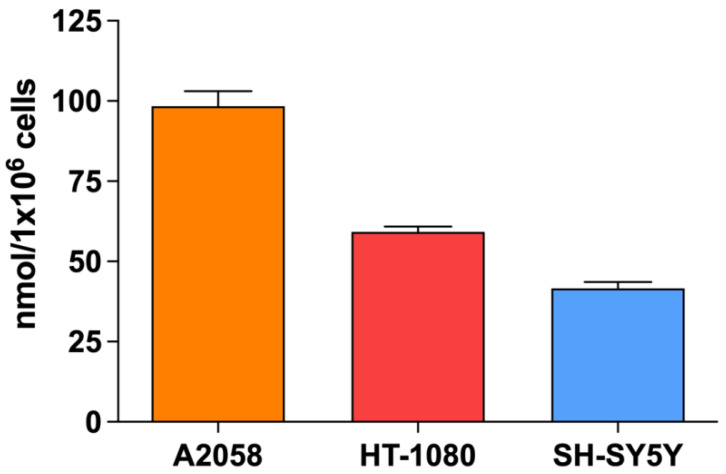
Basal levels of glutathione (GSH) according to cell line, i.e., in untreated cells. GSH levels were determined using the Glutathione Assay Kit (Sigma-Aldrich, St. Louis, MO, USA) and the GSH content (nmol) was normalized to 1 × 10^6^ cells.

As SDT cytotoxicity is mainly caused by SDT-induced oxidative stress, the basal level of reduced glutathione, considered the dominant intracellular antioxidant system, was determined in all cell lines. Observing Figure 1, the A2058 cell line had the highest GSH concentration (98.35 ± 4.73 nmoL/1 × 10^6^ cells) compared to the HT-1080 cell line (59.20 ± 1.69 nmoL/1 × 10^6^ cells) and the SH-SY5 Y cell line (41.64 ± 1.92 nmol/1 × 10^6^ cells).

### 3.2. Evaluation of Ala-Derived PPIX Intracellular Accumulation in A2058, HT-1080 and SH-SY5 Y Cells

**Figure 2:**
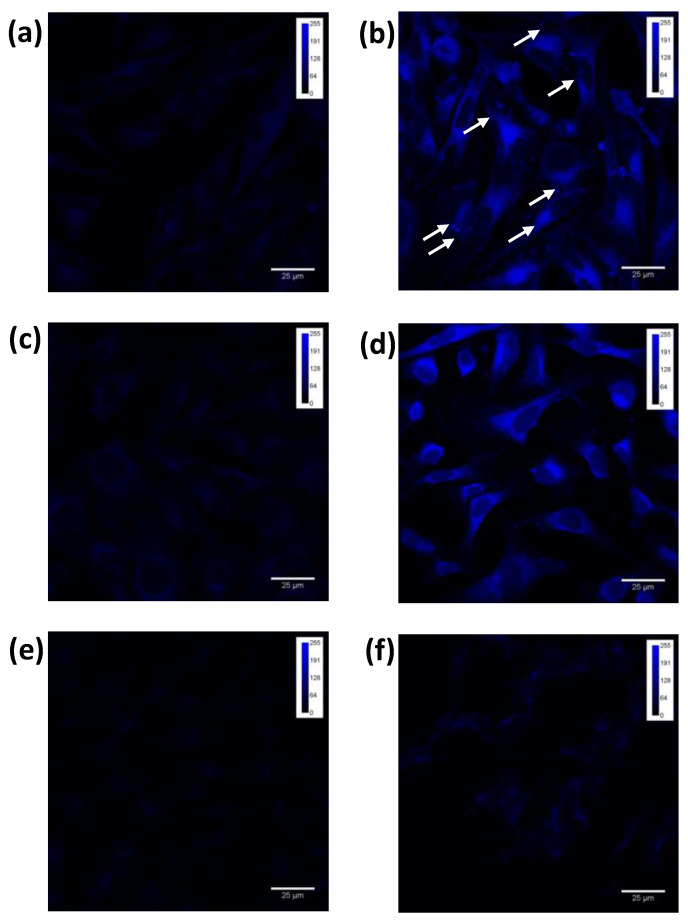
Confocal detection of Ala-derived intracellular PPIX. A2058 (**b**) and HT-1080 (**d**) cells were incubated with 0.45 mM Ala for 12 h and 4 h, respectively; SH-SY5 Y (**f**) cells were incubated with 1.8 mM Ala for 24 h. Panels (**a**,**c**,**e**) represent untreated A2058, HT-1080 and SH-SY5 Y cells, respectively, i.e., cells not incubated with Ala. White arrows indicate the accumulation of PPIX inside melanosomes in A2058 cells. Scale bar: 25 μm.

The intracellular fluorescence of PPIX was determined by flow cytometric analysis to investigate the most appropriate Ala concentration to perform sonodynamic and photodynamic in vitro experiments in A2058, HT-1080 and SH-SY5 Y cell lines. The integrated iMFI ratio of PPIX increased when A2058 cells were exposed to 0.45 mM of Ala for 12 h; HT-1080 cells were exposed to 0.45 mM of Ala for 4 h; and SH-SY5 Y cells were exposed to 1.80 mM of Ala for 24 h (Table 1). Therefore, in SDT and PDT experiments, A2058 and HT-1080 cells were incubated with 0.45 mM Ala for 12 and 4 h, respectively, while SH-SY5 Y cells were incubated with 1.80 mM Ala for 24 h. To confirm the flow cytometric data, the conversion of Ala into PPIX was analysed by confocal imaging in all cell lines incubated with the previous cited concentrations of Ala. As shown in Figure 2, A2058 (4 b) and HT-1080 (4 d) showed a more intense and diffuse PPIX signal into the cytoplasm only after 12 and 4 h of incubation, respectively, with respect to SH-SY5 Y cells (4 f). Moreover, in A2058 PPIX-positive cells (4 b), blue spots inside the cytoplasm were observed, which may indicate PPIX accumulation inside melanosomes.

### 3.3. Effect of SW-Mediated SDT and PDT on Cell Proliferation

**Figure 3:**
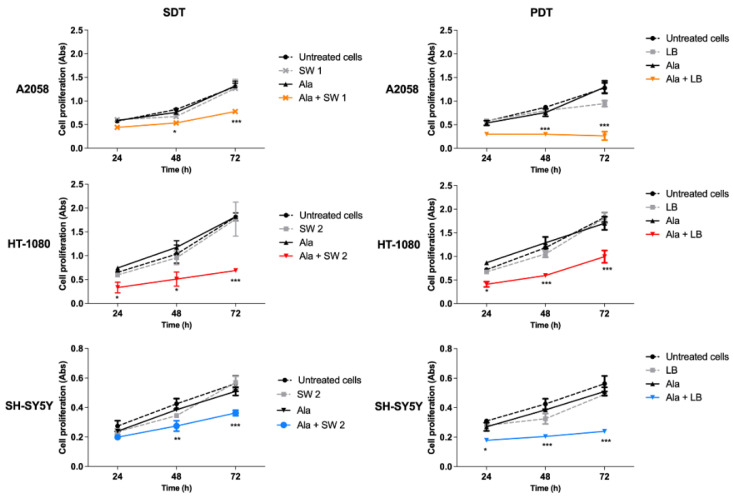
The in vitro effect of SW-mediated SDT and PDT on A2058, HT-1080 and SH-SY5 Y cells. Cells were treated with Ala (0.45 mM for 12 h in A2058 cells, 0.45 mM for 4 h in HT-1080 cells and 1.80 mM for 24 h in SH-SY5 Y cells), shock waves (SW1 0.32 mJ/mm^2^ for 1000 impulses, 4 impulses/s; SW2 0.43 mJ/mm^2^ for 500 impulses, 4 impulses/s) or light beams (LB, 15 mW/cm^2^ for 5 min) and their combination. Statistically significant differences versus untreated cells: * *p* < 0.05, ** *p* < 0.01, *** *p* < 0.001.

SDT and PDT effects on cell proliferation were observed over time, up to 72 h. In all cell lines, SDT induced the highest decrease of cell proliferation at 72 h after treatment (*p* < 0.001) compared to untreated cells (Figure 3). In HT-1080 cells, there was a significant SDT-induced decrease in cell proliferation already at 24 h after treatment (*p* < 0.05). Interestingly, no effect on cell proliferation was observed when all cell lines underwent SW treatment alone. Regarding PDT, a significant decrease in cell proliferation was observed after treatment, already at 24 h in HT-1080 and SH-SY5 Y cells, and at 48 h in A2058 cells, which increased at 72 h (*p* < 0.001), compared to untreated cells.

### 3.4. Evaluation of Cell Death after SW-Mediated SDT and PDT

Investigations on necrotic and apoptotic cell death induced by SDT or PDT was carried out by Sytox^®^ Green and APC-Annexin V staining. Observing Table 2, SDT induced a more pronounced increase in the percentage of apoptotic cells in A2780 (*p* < 0.05), HT-1080 (*p* < 0.001) and SH-SY5 Y (*p* < 0.01) cell lines, along with a significant reduction in the percentage of viable cells, while any significant increase in the percentage of necrotic cells was observed. In addition, a statistically significant increase in the percentage of necrotic A2058 (*p* < 0.001), HT-1080 (*p* < 0.001) and SH-SY5 Y (*p* < 0.01) cells was observed after PDT, along with a significant decrease in the percentage of viable cells, without any significant increase in the percentage of apoptotic cells. These data showed a significant difference in the cell death induced by SDT and PDT, with SDT mainly provoking apoptotic cell death and PDT mainly inducing necrotic cell death.

### 3.5. ROS Production after SW-Mediated SDT and PDT

**Figure 4:**
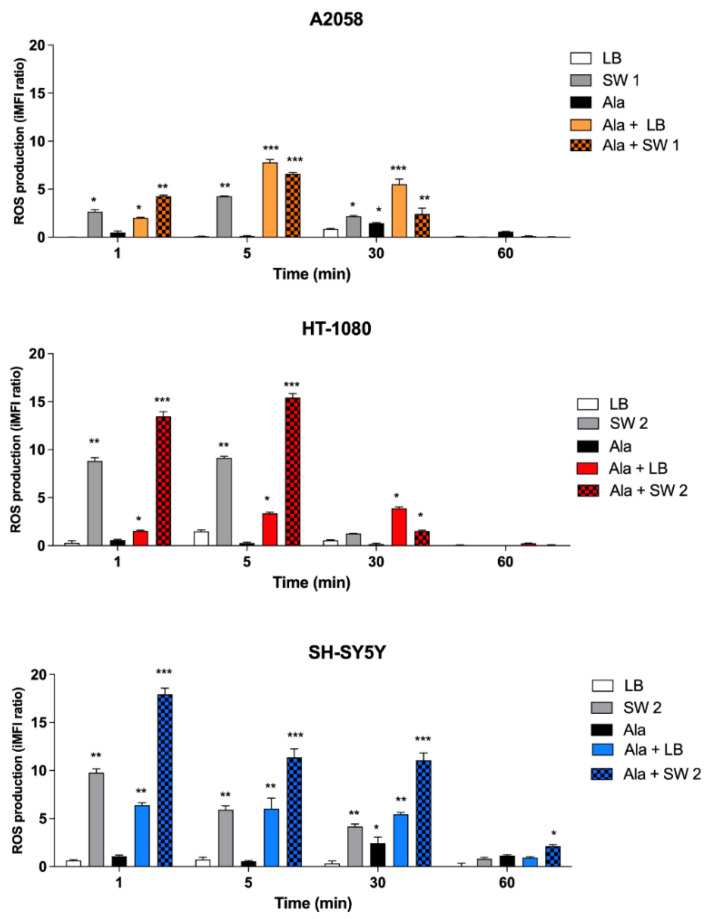
ROS production after SW-mediated SDT and PDT. Untreated cells and cells treated with Ala (0.45 mM for 12 h in A2058 cells, 0.45 mM for 4 h in HT-1080 cells and 1.80 mM for 24 h in SH-SY5 Y cells), shock waves (SW1 0.32 mJ/mm^2^ for 1000 impulses, 4 impulses/s; SW2 0.43 mJ/mm^2^ for 500 impulses, 4 impulses/s) or light beams (LB, 15 mW/cm^2^ for 5 min) and their combination. Statistically significant differences versus untreated cells: * *p* < 0.05 **, *p* < 0.01, *** *p* < 0.001.

As it has been demonstrated that SDT exerts its cytotoxic effect through ROS generation [1,22,23,24], ROS levels were evaluated cytofluorimetrically with a 2′,7′-dichloro-fluorescein diacetate (DCF-DA) probe. As shown in Figure 4, in A2058 cells, SDT and PDT provoked a significant increase in ROS production, which was quite similar over time, with a maximum of ROS production 5 min after both treatments (*p* < 0.001). HT-1080 and SH-SY5 Y cells showed a different pattern of ROS production after SDT and PDT; ROS levels were higher after SDT than after PDT. Moreover, in HT-1080 and SH-SY5 Y cells, we observed a statistically significant ROS production already at 1 min after SDT (*p* < 0.001), followed by a decrease after 60 min. On the other hand, PDT produced a significant ROS production already visible after 1 min of treatment, which was similar over time in both cell lines.

### 3.6. Evaluation of Gene Expression and Cytochrome c Production after SW-Mediated SDT and PDT

**Figure 5:**
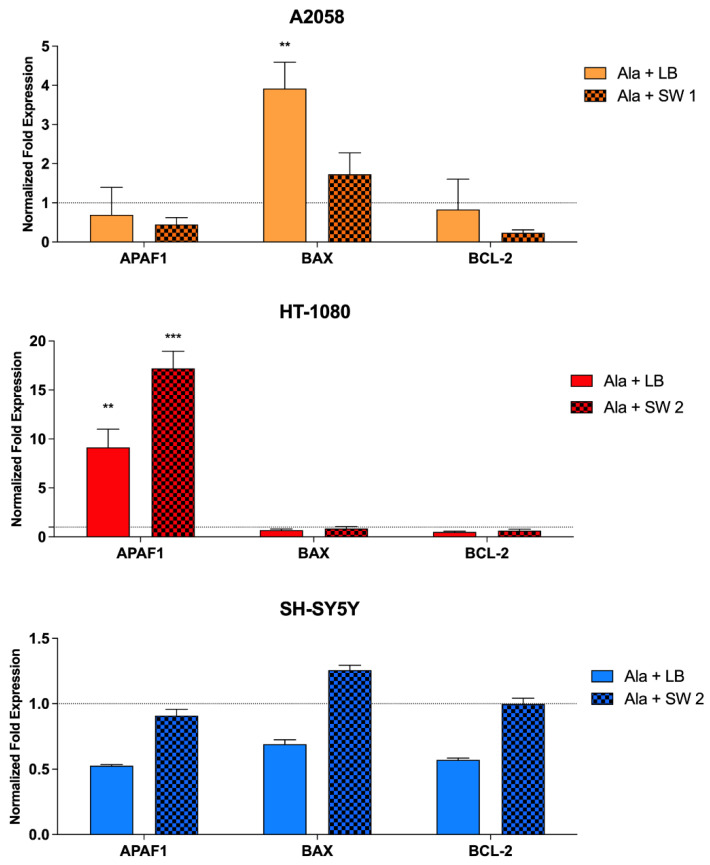
mRNA expression in A2058, HT-1080 and SH-SY5 Y cells after SW-mediated SDT and PDT. *RRN1* (ribosomal RNA 18 S) was used as a reference gene to normalize the data. SDT- and PDT-induced changes in mRNA levels were compared to those of the untreated cells stated as 1 and shown by the dotted line. Statistically significant differences versus untreated cells: ** *p* < 0.01, *** *p* < 0.001.

Real-time RT-PCR analysis was performed to explore the effects that sonodynamic and photodynamic treatments have on the mRNA expression of genes involved in apoptosis and in oxidative stress. At 24 h after SDT or PDT treatments, mRNA gene expression was analysed in all cell lines being investigated. As shown in Figure 5, in A2058 cells, a significant up-regulation of the pro-apoptotic *BAX* gene expression (*p* < 0.01) was observed after PDT compared to untreated cells, while SDT induced an important down-regulation of anti-apoptotic *BCL-2* gene expression compared to untreated cells. In HT-1080 cells, a significant up-regulation of Apoptotic Peptidase Activating Factor 1 (*APAF1*) after SDT (*p* < 0.001) and PDT (*p* < 0.01) compared to untreated cells was observed. In SH-SY5 Y cells, a slight up-regulation of *BAX* gene expression was observed, which was not statistically significant.

**Figure 6:**
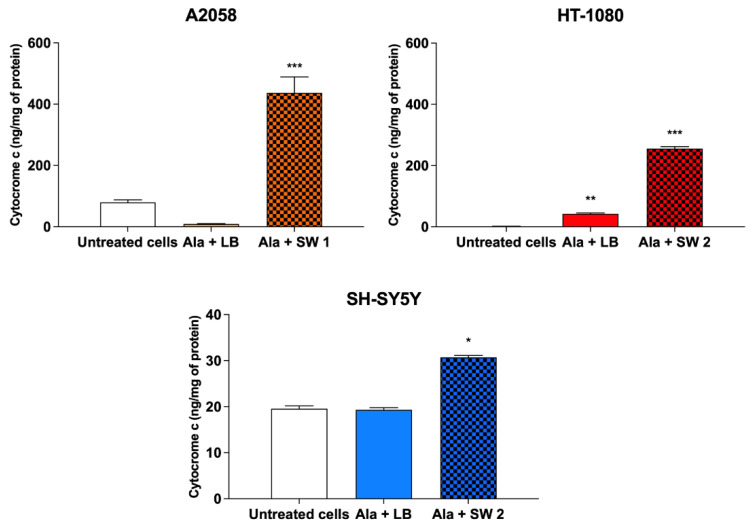
Cytochrome c release after SW-mediated SDT and PDT. Cells were treated with Ala (0.45 mM for 12 h in A2058 cells, 0.45 mM for 4 h in HT-1080 cells and 1.80 mM for 24 h in SH-SY5 Y cells), shock waves (SW1 0.32 mJ/mm^2^ for 1000 impulses, 4 impulses/s; SW2 0.43 mJ/mm^2^ for 500 impulses, 4 impulses/s) or light beams (LB, 15 mW/cm^2^ for 5 min) and their combination. Statistically significant differences versus untreated cells: * *p* < 0.05, ** *p* < 0.01, *** *p* < 0.001.

As cytochrome c release is unique to oxidative stress-mediated apoptosis [25,26,27], its release in cytosolic extracts of cells preincubated with Ala and then exposed to SDT or PDT was evaluated. As observed in Figure 6, a statistically significant increase (*p* < 0.001) in mitochondrial cytochrome c release into the cytosol was observed at 24 h after SDT in all the cell lines investigated, with the highest cytochrome c release in A2058 cells (436.69 ± 51.88, *p* < 0.001). At the same time, only a slight cytochrome c release after PDT was visible in HT-1080 cells (42.35 ± 3.00) (*p* < 0.01). These data support the SDT-apoptotic cell death highlighted by cytofluorimetric analysis (Table 2).

### 3.7. Evaluation of SW-Mediated SDT Effect on DNA Damage

**Figure 7:**
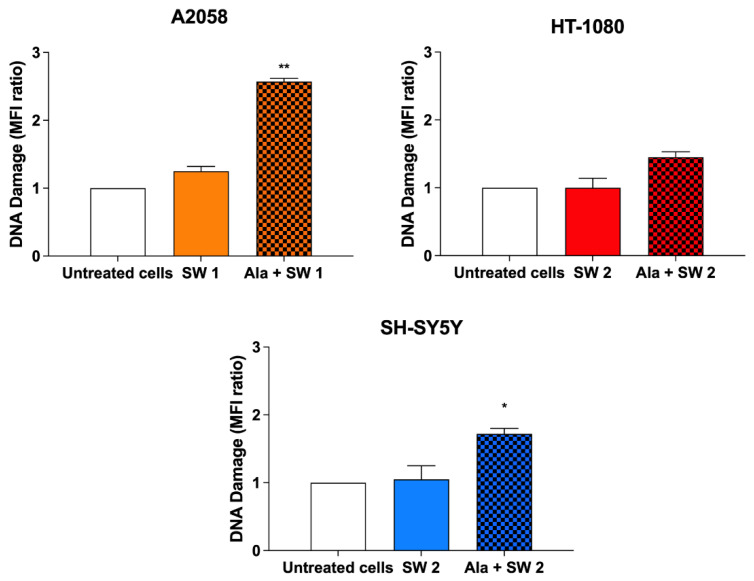
Effect of SW-mediated SDT on DNA damage. Cells were previously incubated with Ala (0.45 mM for 12 h in A2058 cells, 0.45 mM for 4 h in HT-1080 cells and 1.80 mM for 24 h in SH-SY5 Y cells), and then exposed to SW1 (0.32 mJ/mm^2^ for 1000 impulses, 4 impulses/s) or to SW2 (0.43 mJ/mm^2^ for 500 impulses, 4 impulses/s). Statistically significant differences versus untreated cells: * *p* < 0.05, ** *p* < 0.01.

As oxidative stress is known to lead to DNA damage [28], oxidative DNA damage was analysed at 24 h after SDT treatment in all cells using an aldehyde reactive probe. Considering data from Figure 7, a significant increase in DNA damage was detected in A2058 cells (2.57 ± 0.05) (*p* < 0.01) compared to untreated cells. At the same time, the DNA damage in HT-1080 cells was not significant compared to untreated cells, and in SH-SY5 Y cells, there was a slight but statistically significant increase in DNA damage (*p* < 0.05).

### 3.8. SW-Mediated SDT Effect on Spheroid Growth

**Figure 8:**
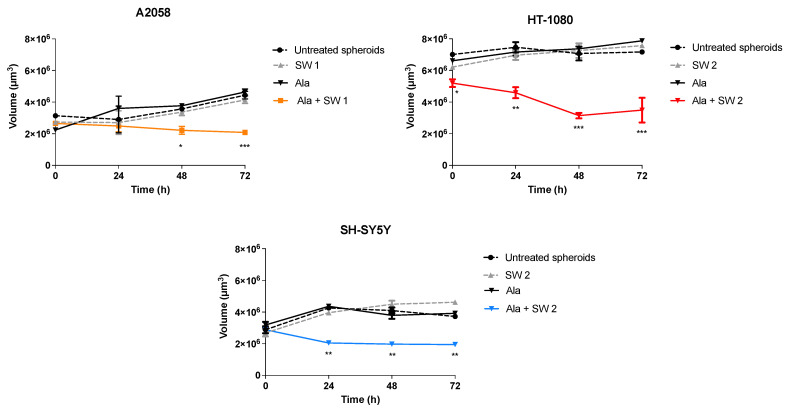
The average volume of A2058, HT-1080 and SH-SY5 Y spheroids after SW-mediated SDT. Spheroids were previously incubated with Ala (0.45 mM for 12 h in A2058 cells, 0.45 mM for 4 h in HT-1080 cells and 1.80 mM for 24 h in SH-SY5 Y cells) and then exposed to SW1 (0.32 mJ/mm^2^ for 1000 impulses, 4 impulses/s) or to SW2 (0.43 mJ/mm^2^ for 500 impulses, 4 impulses/s). Spheroids were also treated with Ala alone and SW1 or SW 2 alone. Statistically significant differences versus untreated spheroids: * *p* < 0.05, ** *p* < 0.01, *** *p* < 0.001.

Three-dimensional models have been introduced in the last 20 years to better illustrate cell distribution in space, and they are more representative of the in vivo organisation, with several improvements in viability, morphology and drug metabolism [18]. Therefore, we focused our attention on SDT effects in the studied cells organized in 3D tumour spheroids. The growth of cell spheroid models was performed after SDT by calculating the spheroid volume through phase contrast images at different time points. Analysis of the growth of spheroids treated with SDT showed a significant reduction, compared to untreated spheroids, in the volume of A2058 spheroids after 48 (*p* < 0.05) and 72 h (*p* < 0.001); in HT-1080 spheroids after 24 (*p* < 0.01), 48 (*p* < 0.001) and 72 h (*p* < 0.001), and in SH-SY5 Y spheroids after 24, 48 and 72 h (*p* < 0.01), without loss of spheroid integrity (Figure 8). Moreover, no differences in spheroid growth after Ala or SW treatment alone were observed, compared to untreated spheroids.

## 4. Discussion

Sonodynamic therapy represents an interesting and innovative anticancer treatment that was initially developed to overcome certain drawbacks of PDT, such as the low penetration of light into the tissues and the low selectivity of the treatment versus the target of interest. The main modality of SDT is the use of US energy to excite a responsive chemical compound, usually hematoporphyrin derivatives. This provokes a chemical reaction which culminates in the formation of cytotoxic singlet oxygen and ROS production [14,29]. SW are specific pulsed US that do not produce the classic thermal effect of US, as the pulse duration of SW is extremely short and generated at low frequencies; therefore, no thermal effect is generated [30]. Shock waves can spread through different substances without important dissipation of energy if the acoustic impedance values of the substances remain almost identical [7]. As the acoustic impedance values of water and soft tissues of the body are similar, SW can propagate through these tissues without any considerable loss of energy. Nevertheless, energy dissipation takes place, and focal mechanical energy can be present at transition sites between tissues that have different values of acoustic impedance. This is probably generated through the shearing stress and cavitation provoked by the reflected waves [31]. 

In this study, we focused our attention on the efficacy of SW in the sonodynamic activation of Ala-derived PPIX on three different human cancer cell lines, namely melanoma (A2058), fibrosarcoma (HT-1080) and neuroblastoma (SH-SY5 Y) cell lines, taking PDT as a reference treatment. Different cell lines were considered as it is well-known that particular cellular and molecular features within cells can influence therapeutic outcomes [19,32]. 

Exposure of Ala-derived PPIX was able to provoke a significant reduction of cancer cell proliferation in all the cell lines investigated as the light exposure of the same sensitizer. Moreover, differences in the extent of the sonodynamic effect on cell proliferation were observed in A2058, HT-1080 and SH-SY5Y cell lines according to their intracellular Ala mitochondrial conversion to PPIX and consequent SW-mediated ROS production. The concentrations and incubation times of Ala required for an optimal conversion to PPIX were higher in SH-SY5 Y cells (1.80 mM Ala for 24 h) compared to A2058 cells (0.45 mM for 12 h) and compared to HT-1080 cells (0.45 mM for 4 h). The different PPIX intracellular accumulation after Ala administration may be influenced by many factors. As it has been reported that mitochondria are the key organelles in PPIX metabolism, it can be assumed that PPIX fluorescence is simply a function of mitochondrial number (i.e., mitochondrial mass), mitochondrial activity or both [33]. To support this, Gibson and colleagues found a correlation between PPIX content and mitochondrial content in proliferating cultures of MCF7 breast cancer and HMESO-1 mesothelioma cells [34]. Moreover, it is known that PPIX accumulation is promoted by low levels of intracellular iron, as iron is crucial for ferrochelatase enzyme activity, responsible for the PPIX conversion to heme [35]. Therefore, we can assume that the lower concentration of PPIX in neuroblastoma cells could be due to the presence of fewer mitochondria compared to melanoma and fibrosarcoma cells. This affirmation is in line with other studies where it has been observed that endogenous PPIX production, in different cell lines, is correlated to mitochondria number following Ala administration [34,36].

The sonodynamic treatment with SW and Ala-derived PPIX induced a statistically significant production of ROS, specifically hydroxyl and peroxyl radicals detected cytofluorimetrically by DCF, confirming the data about the effects on cell proliferation. For example, in HT-1080 fibrosarcoma cells, where we observed an intense accumulation of PPIX inside the cytoplasm, we also observed the highest decrease in cell proliferation after SDT, along with a high level of SDT-induced ROS production and a low level of intracellular GSH. Moreover, the analysis of ROS by the DCF-DA assay showed a higher production of ROS after SDT compared to PDT, especially in fibrosarcoma and neuroblastoma cells. Even though PDT and SDT show similar features because they exploit the synergistic effect of chemical compounds combined with external stimuli, namely light (PDT) and US (SDT), the sensitizer activation by light specifically elicits the singlet oxygen generation as the most important cytotoxic intermediate [37]. Therefore, we can assume that the observed differences in the ROS generation between PDT and SDT relies on differences in the specific radical products derived from the sensitizer with different lifetimes and diffusion distances. To explain how the mechanism of action of SDT differs from that of PDT, it is possible to refer to Moor’s analysis about PDT-stimulated signalling pathways, where it is observed that one of the most important features in the response to PDT is the tissue oxygenation status, and the type of cells involved. Moreover, the consideration on the energy transfer is relevant; for example, close vicinity of the sensitizer to the target is essential when a singlet oxygen is formed, as the latter can propagate only 20 nm during its lifetime [38]. Considering the SDT mechanism of action, we illustrated two different hypotheses on the ROS generation mechanism, which refer to the involvement of acoustic cavitation and sonolysis and to the sonoluminescence phenomenon [39,40]. 

Concerning the type of cell death induced by SDT with SW and Ala-derived PPIX, flow cytometric analysis revealed a predominant apoptotic pathway in all the cell lines investigated. The observed SDT-induced cancer cell apoptosis is in line with other studies on SDT in colorectal cancer cells [41] and in anaplastic thyroid cancer cells [15]. On the other hand, the photodynamic activation of Ala-derived PPIX caused necrotic cancer cell death rather than apoptotic cancer cell death in all cell lines. These data are consistent with several studies which report that PDT can induce necrosis at several levels, leading to cell plasma membrane integrity loss [42]. In particular, Coupienne and colleagues were able to identify a receptor-interacting protein 3 (RIP3) as a key element of the caspase-independent form in the induction of glioblastoma cell necrosis in response to PDT with Ala [43]. 

The differences in cell death pathways observed between SDT (mainly apoptotic), and PDT (mainly necrotic) can be explained by the different molecular pathway activation. It has been reported in the literature that apoptosis induced by SDT could be due to three main mechanisms, namely (i) the changeable BCL-2 family protein expression levels and massive ROS production to reduce mitochondrial membrane potential; (ii) the increased presence of calcium ions in the mitochondrial membrane; (iii) the up-regulation of intracellular FAS/FASL expression provoking intracellular caspase-8 and caspase-3 increases [44]. On the other hand, PDT can provoke cell death by either the apoptotic or necrotic pathway, even if, in many cases, necrosis is the preferred mechanism, associated with loss of membrane integrity and ATP depletion upon photoactivation [45].

Induction of cell death was also supported by gene expression evaluation, where a statistically significant increase in the pro-apoptotic gene *BAX* was noticed in A2058 cells after photodynamic treatment, while in HT-1080 cells, both treatments determined a statistically significant increase in *APAF1* gene expression. The same pathway at the protein expression level has also been demonstrated by Li and colleagues, by exposing THP-1 macrophages to a hypericin-mediated sonodynamic treatment and by observing progressive translocation of the BAX protein from the cytosol to the mitochondria, increasing the BAX/BCL-2 ratio [46]. To support the observed apoptotic cell death induced by SW-mediated SDT, we also highlighted a significant release of cytochrome c in all the cell lines after SDT with SW and Ala-derived PPIX, unlike that observed in the same cell lines treated with PDT. Furthermore, in all the cell lines, it was also possible to highlight significant DNA damage after SDT with SW and Ala-derived PPIX.

Finally, to confirm the in vitro data, on 2D cancer cell cultures, A2058, HT-1080 and SH-SY5 Y spheroids were developed as they represent a more realistic network of cell–cell and cell–matrix interactions compared to 2D cultures that do not accurately reflect the in vivo cellular environment [47,48]. The most pronounced decrease in spheroid growth over time was observed in HT-1080 spheroids compared to A2058 and SH-SY5 Y spheroids, such as that observed in 2D cell cultures of the same cell lines. It should be noted that the integrity of 3D cell cultures of all cell lines was unaffected by their exposure to SW only. 

These data highlight the synergic activity between Ala-derived PPIX and SW and are in line with Canaparo’s previous work, where a statistically significant reduction of neuroblastoma spheroids was observed after sonodynamic exposure of meso-tetrakis (4-sulfonatophenyl) porphyrin [13]. Moreover, a significant sonodynamic activity over 3D structures has also been demonstrated by Logan and colleagues who incubated MCF-7 spheroids with microbubbles loaded with doxorubicin, paclitaxel and rose bengal, and then exposed them to US treatment [49]. The results obtained in this study show that the anticancer effect of SDT is remarkably augmented by SW, thus suggesting that SW-mediated SDT can be developed as an innovative and valid approach for cancer treatment.

## Figures and Tables

**Table 1:** Ala-derived PPIX intracellular accumulation in A2058, HT-1080 and SH-SY5 Y cells.

**A2058–Ala [mM]**	**PPIX at 12 h**	
	**Cell Population (%)**	**iMFI Ratio**
**0.45**	90.89 ± 0.16	57.71 ± 0.77
**1.5**	90.73 ± 1.03	57.39 ± 1.96
**3.0**	89.55 ± 0.64	45.92 ± 1.29
**HT-1080–Ala [mM]**	**PPIX at 4 h**	
	**Cell Population (%)**	**iMFI ratio**
**0.45**	85.43 ± 0.80	37.20 ± 1.13
**1.5**	84.37 ± 0.90	35.99 ± 0.72
**3.0**	83.54 ± 0.76	37.02 ± 1.44
**SH-SY5 Y–Ala [mM]**	**PPIX at 24 h**	
	**Cell Population (%)**	**iMFI ratio**
**0.45**	54.36 ± 0.19	8.28 ± 1.29
**0.9**	69.73 ± 0.99	38.77 ± 1.78
**1.8**	76.23 ± 0.92	53.89 ± 1.30
**3.6**	49.38 ± 0.16	10.90 ± 0.83

**Table 2:** Flow cytometric evaluation of A2058, HT-1080 and SH-SY5 Y cell death after SW-mediated SDT and PDT.

**A2058 Cells**			
**Cell Treatment 48 h**	**Viable Cells (%)**	**Apoptotic Cells (%)**	**Necrotic Cells (%)**
**Untreated cells**	81.62 ± 0.54	8.66 ± 0.48	9.94 ± 0.09
**SW 1**	81.73 ± 0.38	9.58 ± 0.60	8.55 ± 0.64
**LB**	80.26 ± 1.05	6.80 ± 1.00	14.59 ± 0.60
**Ala**	72.27 ± 0.38	10.91 ± 0.13	16.02 ± 0.02
**Ala + SW 1**	74.58 ± 0.81	17.51 ± 1.21 *	8.60 ± 0.57
**Ala + LB**	26.58 ± 0.59 ***	7.56 ± 0.62	65.88 ± 0.18 ***
**HT-1080 cells**			
**Cell treatment 24 h**	**Viable cells (%)**	**Apoptotic cells (%)**	**Necrotic cells (%)**
**Untreated cells**	79.18 ± 1.17	6.76 ± 0.35	15.19 ± 1.15
**SW 2**	74.44 ± 2.03	10.93 ± 0.11	13.13 ± 1.24
**LB**	77.22 ± 1.11	15.03 ± 0.67 *	9.11 ± 0.55
**Ala**	81.89 ± 1.25	7.11 ± 0.16	8.79 ± 0.41 *
**Ala + SW 2**	65.90 ± 1.27 *	21.46 ± 0.76 ***	11.19 ± 0.26
**Ala + LB**	60.04 ± 1.36 *	10.18 ± 1.17	27.94 ± 0.73 **
**SH-SY5 Y cells**			
**Cell treatment 24 h**	**Viable cells (%)**	**Apoptotic cells (%)**	**Necrotic cells (%)**
**Untreated cells**	87.14 ± 1.22	7.89 ± 0.16	5.89 ± 0.16
**SW 2**	85.31 ± 0.98	9.63 ± 0. 53	6.25 ± 0.36
**LB**	84.84 ± 1.18	7.12 ± 0.16	6.95 ± 0.08
**Ala**	89.22 ± 1.10	6.42 ± 0.59	5.04 ± 0.66
**Ala + SW 2**	72.99 ± 1.40	15.97 ± 1.36 **	8.96 ± 0.06
**Ala + LB**	53.84 ± 0.23 ***	10.69 ± 0.98	34.31 ± 0.43 ***

Statistically significant difference versus untreated cells: * *p* < 0.05, ** *p* < 0.01, *** *p* < 0.001.

## Data Availability

Data is contained within the article.

## References

[1] Choi V., Rajora M.A., Zheng G. (2020). Activating Drugs with Sound: Mechanisms Behind Sonodynamic Therapy and the Role of Nanomedicine. Bioconjug. Chem..

[2] Lafond M., Yoshizawa S., Umemura S.-I. (2018). Sonodynamic Therapy: Advances and Challenges in Clinical Translation. J. Ultrasound Med..

[3] Shibaguchi H., Tsuru H., Kuroki M., Kuroki M. (2011). Sonodynamic cancer therapy: A non-invasive and repeatable approach using low-intensity ultrasound with a sonosensitizer. Anticancer Res..

[4] Villeneuve L., Alberti L., Steghens J.-P., Lancelin J.-M., Mestas J.-L. (2009). Assay of hydroxyl radicals generated by focused ultrasound. Ultrason. Sonochem..

[5] Gielen B., Jordens J., Janssen J., Pfeiffer H., Wevers M., Thomassen L., Braeken L., Van Gerven T. (2014). Characterization of stable and transient cavitation bubbles in a milliflow reactor using a multibubble sonoluminescence quenching technique. Ultrason. Sonochem..

[6] Serpe L., Foglietta F., Canaparo R. (2012). Nanosonotechnology: The next challenge in cancer sonodynamic therapy. Nanotechnol. Rev..

[7] Wan G.-Y., Liu Y., Chen B.-W., Liu Y.-Y., Wang Y., Zhang N. (2016). Recent advances of sonodynamic therapy in cancer treatment. Cancer Biol. Med..

[8] Sun X., Xu H., Shen J., Guo S., Shi S., Dan J., Tian F., Tian Y., Tian Y. (2015). Real-time detection of intracellular reactive oxygen species and mitochondrial membrane potential in THP-1 macrophages during ultrasonic irradiation for optimal sonodynamic therapy. Ultrason. Sonochem..

[9] Giuntini F., Foglietta F., Marucco A.M., Troia A., Dezhkunov N.V., Pozzoli A., Durando G., Fenoglio I., Serpe L., Canaparo R. (2018). Insight into ultrasound-mediated reactive oxygen species generation by various metal-porphyrin complexes. Free Radic. Biol. Med..

[10] Foglietta F., Canaparo R., Francovich A., Arena F., Civera S., Cravotto G., Frairia R., Serpe L. (2015). Sonodynamic treatment as an innovative bimodal anticancer approach: Shock wave-mediated tumor growth inhibition in a syngeneic breast cancer model. Discov. Med..

[11] Maruyama M., Asano T., Uematsu T., Nakagohri T., Hasegawa M., Miyauchi H., Iwashita C., Isono K. (1995). Enhancement of the Antitumor Effect by Combined Use of High-energy Shock Waves and ATX-70. Jpn. J. Cancer Res..

[12] Wess O.J., Talati J., Tiselius H.G., Albala D., Ye Z. (2012). Physics and Technique of Shock Wave Lithotripsy (SWL). Urolithiasis.

[13] Serpe L., Canaparo R., Varchi G., Ballestri M., Foglietta F.F., Sotgiu G., Guerrini A., Francovich A., Civera P., Frairia R. (2013). Polymeric nanoparticles enhance the sonodynamic activity of meso-tetrakis (4-sulfonatophenyl) porphyrin in an in vitro neuroblastoma model. Int. J. Nanomed..

[14] Serpe L., Canaparo R., Berta L., Bargoni A., Zara G.P., Frairia R. (2011). High Energy Shock Waves and 5-Aminolevulinic for Sonodynamic Therapy: Effects in a Syngeneic Model of Colon Cancer. Technol. Cancer Res. Treat..

[15] Catalano M.G., Costantino L., Fortunati N., Bosco O., Pugliese M., Boccuzzi G., Berta L., Frairia R. (2007). High Energy Shock Waves Activate 5′-Aminolevulinic Acid and Increase Permeability to Paclitaxel: Antitumor Effects of a New Combined Treatment on Anaplastic Thyroid Cancer Cells. Thyroid.

[16] Kapałczyńska M., Kolenda T., Przybyła W., Zajączkowska M., Teresiak A., Filas V., Ibbs M., Bliźniak R., Łuczewski L., Lamperska K. (2018). 2D and 3D cell cultures—A comparison of different types of cancer cell cultures. Arch. Med. Sci..

[17] Pape J., Emberton M., Cheema U. (2021). 3D Cancer Models: The Need for a Complex Stroma, Compartmentalization and Stiffness. Front. Bioeng. Biotechnol..

[18] Foglietta F., Canaparo R., Muccioli G., Terreno E., Serpe L. (2020). Methodological aspects and pharmacological applications of three-dimensional cancer cell cultures and organoids. Life Sci..

[19] Qin S., Jiang J., Lu Y., Nice E.C., Huang C., Zhang J., He W. (2020). Emerging role of tumor cell plasticity in modifying therapeutic response. Signal Transduct. Target. Ther..

[20] Wang X., Fang H., Huang Z., Shang W., Hou T., Cheng A., Cheng H. (2013). Imaging ROS signaling in cells and animals. Klin. Wochenschr..

[21] Bustin S.A., Benes V., Garson J.A., Hellemans J., Huggett J., Kubista M., Mueller R., Nolan T., Pfaffl M.W., Shipley G.L. (2009). The MIQE Guidelines: Minimum Information for Publication of Quantitative Real-Time PCR Experiments. Clin. Chem..

[22] Rosenthal I., Sostaric J.Z., Riesz P. (2004). Sonodynamic therapy––a review of the synergistic effects of drugs and ultrasound. Ultrason. Sonochem..

[23] Fu J., Li T., Zhu Y., Hao Y. (2019). Ultrasound-Activated Oxygen and ROS Generation Nanosystem Systematically Modulates Tumor Microenvironment and Sensitizes Sonodynamic Therapy for Hypoxic Solid Tumors. Adv. Funct. Mater..

[24] Jin Y., Zhou Q., Geng J., Meng Q., Wei Z., Ding M., Zhou J., Zeng Y., Cao W., Liu F. (2021). Sonodynamic Effects of a Novel Ether-Group Modified Porphyrin Derivative Combined with Pulsed Low-Intensity Ultrasound on PC-9 Cells. Front. Pharmacol..

[25] Castellano A.G., Díaz-Quintana A., Pérez-Mejías G., Elena-Real C.A., González-Arzola K., García-Mauriño S.M., De la Rosa M.A., Díaz-Moreno I. (2018). Oxidative stress is tightly regulated by cytochrome c phosphorylation and respirasome factors in mitochondria. Proc. Natl. Acad. Sci. USA.

[26] Jiang X., Wang X. (2004). Cytochrome C-Mediated Apoptosis. Annu. Rev. Biochem..

[27] Garrido C., Galluzzi L., Brunet M., Puig P.E., Didelot C., Kroemer G. (2006). Mechanisms of cytochrome c release from mitochondria. Cell Death Differ..

[28] Sun Y., Wang H., Zhang K., Liu J., Wang P., Wang X., Liu Q. (2021). Correction: Sonodynamic therapy induces oxidative stress, DNA damage and apoptosis in glioma cells. RSC Adv..

[29] Noodt B.B., Berg K.S., Stokke T., Peng Q., Nesland J.M. (1996). Apoptosis and necrosis induced with light and 5-aminolaevulinic acid-derived protoporphyrin IX. Br. J. Cancer.

[30] Cheing G.L.Y., Chang H. (2003). Extracorporeal Shock Wave Therapy. J. Orthop. Sports Phys. Ther..

[31] Kato K., Fujimura M., Nakagawa A., Saito A., Ohki T., Takayama K., Tominaga T. (2007). Pressure-dependent effect of shock waves on rat brain: Induction of neuronal apoptosis mediated by a caspase-dependent pathway. J. Neurosurg..

[32] Baghban R., Roshangar L., Jahanban-Esfahlan R., Seidi K., Ebrahimi-Kalan A., Jaymand M., Kolahian S., Javaheri T., Zare P. (2020). Tumor microenvironment complexity and therapeutic implications at a glance. Cell Commun. Signal..

[33] Krieg R.C., Messmann H., Rauch J., Seeger S., Knuechel R. (2002). Metabolic characterization of tumor cell-specific proto-porphyrin IX accumulation after exposure to 5-aminolevulinic acid in human colonic cells. Photochem. Photobiol..

[34] Gibson S.L., Nguyen M.L., Havens J.J., Barbarin A., Hilf R. (1999). Relationship of δ-Aminolevulinic Acid-Induced Protoporphyrin IX Levels to Mitochondrial Content in Neoplastic Cells in Vitro. Biochem. Biophys. Res. Commun..

[35] Wyld L., Burn J.L., Reed M., Brown N. (1997). Factors affecting aminolaevulinic acid-induced generation of protoporphyrin IX. Br. J. Cancer.

[36] Gibbs S.L., Chen B., O’Hara J.A., Hoopes P.J., Hasan T., Pogue B.W. (2006). Protoporphyrin IX Level Correlates with Number of Mitochondria, But Increase in Production Correlates with Tumor Cell Size. Photochem. Photobiol..

[37] Moan J., Berg K. (1991). The photodegradation of porphyrins in cells can be used to estimate the lifetime of singlet oxygen. Photochem. Photobiol..

[38] Moor A.C. (2000). Signaling pathways in cell death and survival after photodynamic therapy. J. Photochem. Photobiol. B Biol..

[39] Beguin E., Shrivastava S., Dezhkunov N.V., McHale A.P., Callan J.F., Stride E. (2019). Direct Evidence of Multibubble Sonoluminescence Using Therapeutic Ultrasound and Microbubbles. ACS Appl. Mater. Interfaces.

[40] Costley D., Mc Ewan C., Fowley C., McHale A.P., Atchison J., Nomikou N., Callan J.F. (2015). Treating cancer with sonodynamic therapy: A review. Int. J. Hyperth..

[41] Shen Y., Ou J., Chen X., Zeng X., Huang L., Pi Z., Hu Y., Chen S., Chen T. (2020). An in vitro study on sonodynamic treatment of human colon cancer cells using sinoporphyrin sodium as sonosensitizer. Biomed. Eng. Online.

[42] Zong W.-X., Thompson C.B. (2006). Necrotic death as a cell fate. Genes Dev..

[43] Coupienne I., Fettweis G., Rubio N., Agostinis P., Piette J. (2011). 5-ALA-PDT induces RIP3-dependent necrosis in glioblastoma. Photochem. Photobiol. Sci..

[44] Rengeng L., Qianyu Z., Yuehong L., Zhongzhong P., Libo L. (2017). Sonodynamic therapy, a treatment developing from photodynamic therapy. Photodiagnosis Photodyn. Ther..

[45] Donohoe C., Senge M.O., Arnaut L., da Silva L.C.G. (2019). Cell death in photodynamic therapy: From oxidative stress to anti-tumor immunity. Biochim. Biophys. Acta Rev Cancer.

[46] Yang L., Li X., Gao L., Zheng L., Kou J., Zhu X., Jiang Y., Zhong Z., Dan J., Xu H. (2015). The efficacy and mechanism of apoptosis induction by hypericin-mediated sonodynamic therapy in THP-1 macrophages. Int. J. Nanomed..

[47] Rimann M., Graf-Hausner U. (2012). Synthetic 3D multicellular systems for drug development. Curr. Opin. Biotechnol..

[48] Griffith L.G., Swartz M.A. (2006). Capturing complex 3D tissue physiology in vitro. Nat. Rev. Mol. Cell Biol..

[49] Logan K., Foglietta F., Nesbitt H., Sheng Y., McKaig T., Kamila S., Gao J., Nomikou N., Callan B., McHale A.P. (2019). Targeted chemo-sonodynamic therapy treatment of breast tumours using ultrasound responsive microbubbles loaded with paclitaxel, doxorubicin and Rose Bengal. Eur. J. Pharm. Biopharm..

